# Influence of Bariatric Surgery on Gut Microbiota Composition and Its Implication on Brain and Peripheral Targets

**DOI:** 10.3390/nu16071071

**Published:** 2024-04-05

**Authors:** Sevag Hamamah, Andras Hajnal, Mihai Covasa

**Affiliations:** 1 Department of Basic Medical Sciences, College of Osteopathic Medicine, Western University of Health Sciences, Pomona, CA 9176, USA; sevag.hamamah@westernu.edu; 2 Department of Neural and Behavioral Sciences, College of Medicine, The Pennsylvania State University, Hershey, PA 17033, USA; ahajnal@psu.edu; 3 Department of Biomedical Sciences, College of Medicine and Biological Science, University of Suceava, 7200229 Suceava, Romania

**Keywords:** Roux-en-Y gastric bypass, sleeve gastrectomy, gut microbiome, satiety, dopamine

## Abstract

Obesity remains a significant global health challenge, with bariatric surgery remaining as one of the most effective treatments for severe obesity and its related comorbidities. This review highlights the multifaceted impact of bariatric surgery beyond mere physical restriction or nutrient malabsorption, underscoring the importance of the gut microbiome and neurohormonal signals in mediating the profound effects on weight loss and behavior modification. The various bariatric surgery procedures, such as Roux-en-Y gastric bypass (RYGB) and sleeve gastrectomy (SG), act through distinct mechanisms to alter the gut microbiome, subsequently impacting metabolic health, energy balance, and food reward behaviors. Emerging evidence has shown that bariatric surgery induces profound changes in the composition of the gut microbiome, notably altering the *Firmicutes*/*Bacteroidetes* ratio and enhancing populations of beneficial bacteria such as *Akkermansia*. These microbiota shifts have far-reaching effects beyond gut health, influencing dopamine-mediated reward pathways in the brain and modulating the secretion and action of key gut hormones including ghrelin, leptin, GLP-1, PYY, and CCK. The resultant changes in dopamine signaling and hormone levels contribute to reduced hedonic eating, enhanced satiety, and improved metabolic outcomes. Further, post-bariatric surgical effects on satiation targets are in part mediated by metabolic byproducts of gut microbiota like short-chain fatty acids (SCFAs) and bile acids, which play a pivotal role in modulating metabolism and energy expenditure and reducing obesity-associated inflammation, as well as influencing food reward pathways, potentially contributing to the regulation of body weight and reduction in hedonic eating behaviors. Overall, a better understanding of these mechanisms opens the door to developing non-surgical interventions that replicate the beneficial effects of bariatric surgery on the gut microbiome, dopamine signaling, and gut hormone regulation, offering new avenues for obesity treatment.

## 1. Introduction

Bariatric surgery continues to serve as one of the most efficient treatment strategies to promote significant weight loss in individuals with severe obesity (body mass index; BMI > 35 kg/m^2^), while also aiding in the mitigation of related complications, including, but not limited to type 2 diabetes mellitus, cardiovascular disease, and non-alcoholic liver disease [1]. In the United States alone, it is projected that by 2030, the prevalence of obesity (BMI > 30 kg/m^2^) in adults will reach nearly one in two individuals, while the prevalence of severe obesity will be nearly one in four [2,3]. Worldwide, obesity also consistently contributes to morbidity and mortality with a projected global prevalence of over one billion in the next 5–10 years [4]. In more than 70 countries, the prevalence of childhood obesity has doubled with an estimated 254 million occurrences under the age of 18 [5]. Therefore, bariatric surgery will continue to play an integral role in combating weight gain for years to come, and identifying potential contributors in these processes that sustain weight loss will help augment this treatment modality. Presently, the mechanisms behind the weight loss achieved through bariatric surgery are well-documented and include reducing the capacity for food intake to promote early feelings of fullness, limiting the surface area available for nutrient absorption, and modifying gut hormones that affect feelings of satiety and the neural regulation of energy balance [6,7,8].

In recent years, it has been demonstrated that bariatric surgery impacts the composition of gut microbiota [9]. These shifts in microbial populations have been directly linked to changes in the neural and peripheral hormones that control food intake [9,10]. The human gastrointestinal tract harbors trillions of symbiotic bacterial species, collectively termed gut microbiota, which provide numerous health benefits when adequate microbial diversity and abundance are achieved [11,12]. However, in states of dysbiosis, changes in the composition profile of gut microbiota and its metabolic byproducts can contribute to the onset of pathogenic conditions [13,14]. There are six main phyla of gut bacteria that include Firmicutes, Bacteroidetes, Verrucomicrobia, Actinobacteria, Euryarchaeota, and Proteobacteria, with over 90% of the total composition being composed of Firmicutes and Bacteroidetes [15]. Due to the high proportion of these two phyla, they have been extensively studied, and significant research has shown that an increase in the *Firmicutes*/*Bacteroidetes* ratio correlates with low-grade systemic inflammatory conditions including obesity and related sequelae [16,17]. Bariatric surgery has been shown to normalize this ratio as well as cause taxonomical shifts that serve a role in promoting weight loss [18]. Interestingly, findings have also shown that pre-operative gut microbiota can have an impact on bariatric surgery outcomes with the *Prevotella*/*Bacteroides* ratio being significantly higher in those who respond to surgical interventions [19]. The contribution of gut microbiota to satiety and behavioral processes related to energy balance post-bariatric surgery is believed to stem from modulation of the vagal signaling, production of short-chain fatty acids (SCFA), changes in bile acid metabolism, as well as anti-inflammatory effects [8,20,21]. These changes are conveyed via the microbiota–gut–brain axis that detects shifts in the microbiota and its metabolites, transmitting this information bidirectionally to modify brain chemistry, neurotransmitters, and neuropeptides [22,23]. Alternatively, microbiota and its metabolites confer significant local influence in the periphery through altering gut permeability and inflammation as well as enteroendocrine cells signaling through the release of gut hormones such as glucagon-like peptide 1 (GLP-1), peptide YY (PYY), and cholecystokinin (CCK) [24]. As such, gut microbiota plays an important role in post-surgical weight loss.

This review focuses on the impact of bariatric surgery on gut microbiota, including similarities and differences in microbiota-related effects based on the two most common bariatric surgeries, Roux-en-Y Gastric Bypass (RYGB) and Sleeve Gastrectomy (SG). In the process, we review the physiologic mechanisms by which gut microbiota and their metabolites modulate regulatory pathways influencing satiety, including vagal afferents, inflammation, short-chain fatty acids, and bile acids. The bacteria taxonomical changes following bariatric surgery and the associated effects on neural and peripheral targets, such as the agouti regulatory protein (AgRP), neuropeptide-Y (NPY), and proopiomelanocortin (POMC), as well as gut hormones, ghrelin, leptin, CCK, GLP-1, and PYY, are also discussed. Lastly, we link bariatric surgery-induced alterations in the microbiota–gut–brain axis to regulatory mechanisms mediating the homeostatic and non-homeostatic control of eating. Overall, this review aims to better elucidate the link between bariatric surgery, intestinal microbes, and satiety peptides to promote a deeper understanding of the contribution of gut microbiota in therapeutic interventions for obesity.

## 2. Bariatric Surgery and Changes in Gut Microbiota

Currently, there are two common types of bariatric surgery that together account for a total of approximately 200,000 procedures in the United States alone [25]. In a recent survey by the International Federation for the Surgery of Obesity and Metabolic Disorders (IFSO), approximately 600,000 bariatric surgical and endoluminal surgeries were performed globally in 2021 [26]. These include various procedural subtypes, such as SG and RYGB, with SG currently being the more common of the two procedures [25]. RYGB and SG have been shown to have great efficacy in promoting weight loss and generally positive effects on altering gut microbiota [9], primarily through improving the *Firmicutes*/*Bacteroidetes* ratio [18,27]. Similarly, both procedures have been shown to increase the microbiota Shannon’s diversity index and gene richness [28]. Commonly, an increase in gut microbial diversity and species richness indicates a healthier gut phenotype, while the opposite correlates with dysbiosis and the development of pathogenic conditions [29]. While there are broad commonalities in gut metagenomics, specific alterations in gut microbiota have been identified as unique to particular surgical procedures [30]. Furthermore, rises in the Shannon’s diversity index can be attributed, to some extent, to variations in the relative abundances of different microbial genera [28]. Many factors are known to contribute to alterations in post-surgical gut microbiota, including, but not limited to, the mechanical restriction of food intake, changes in nutrient absorption, food choices, anatomical modifications, and changes in intestinal pH [31]. These differences within the taxonomical shifts in microbial composition between the two bariatric surgical procedures will be discussed in the following subsections.

### 2.1. Roux-en-Y Gastric Bypass (RYGB) and Gut Microbiota

RYGB surgery is accomplished through the creation of a small gastric pouch with the formation of the Roux limb, which is an anastomosis between the gastric pouch and the jejunum [32]. This gastrojejunal anastomosis bypasses the proximal small intestine, a major nutrient digestion and absorption site, further contributing to weight loss in addition to mechanical restriction through the reduction in the overall size of the proximal stomach [32]. The RYGB procedure typically results in a loss of about 70% of excess body weight, or approximately 30% to 35% of total body weight [33]. Due to the bimodal restrictive and malabsorptive effects, RYGB has been shown to promote increased weight loss of up to 20% more than SG at 24 months, although it is considered a more difficult procedure [34]. Similarly, the RYGB procedure was shown to have a greater estimated percentage of estimated weight loss sustained over a period of 10 years when compared to SG [35].

**Figure 1:**
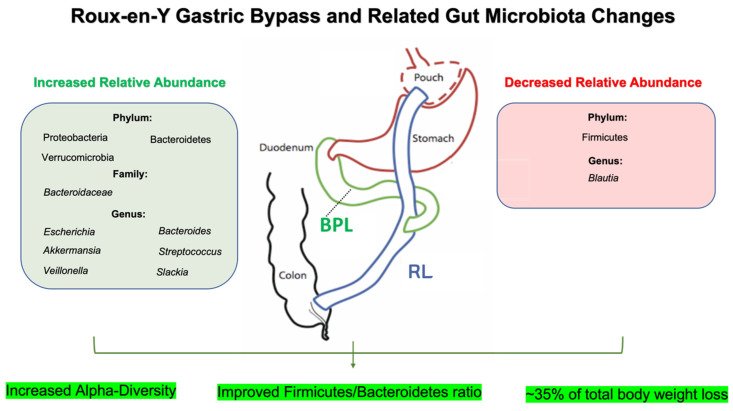
Roux-en-Y Gastric Bypass (RYGB) and Related Gut Microbiota Changes. The middle graph depicts the RYGB procedure. A gastric pouch is created and attached to a segment of the jejunum creating a gastrojejunostomy. The remnant stomach connects with the biliopancreatic limb, which receives bile, pancreatic enzymes, and other secretions. The biliopancreatic limb is anastomosed to the roux-limb. Following RYGB, relative abundances of phyla Proteobacteria, Bacteroidetes, and Verrucomicrobia are increased, while Firmicutes is decreased. Family *Bacteroidaceae* is increased. Genera *Escherichia*, *Akkermansia*, *Veillonella*, *Bacteroides*, *Streptococcus*, and *Slackia* are characteristically increased, while *Blautia* is decreased. Overall, RYGB increases alpha-diversity, improves the *Firmicutes*/*Bacteroidetes* ratio, and confers an average of 35% total body weight loss. Abbreviations: BPL, Biliary-Pancreatic Limb; RL, Roux-Limb.

Although mechanical restriction and malabsorption play a significant role in weight loss following bariatric surgery, the full extent of its effects can also be partially attributed to changes in regulatory signaling via gut–brain communication, initiated by conserved changes in gut microbial composition [9,10]. It has been shown that RYGB increases alpha-diversity and improves the *Firmicutes*/*Bacteroidetes* ratio, but to a lesser extent compared to SG [36]. These post-RYGB changes were associated with increases in *Escherichia* and *Bacteroides*. These findings are consistent with three other studies that showed post-RYGB increases in concentrations of the phylum Proteobacteria and family *Bacteroidaceae*, with concurrent decreases in the phylum Firmicutes [10,37,38] (Figure 1). Interestingly, it has also been shown that up to 37% of increased bacteria following RYGB belong to the phylum Proteobacteria, with these alterations in gut microbiota being accompanied with positive effects on genes associated with white adipose tissue expression [39]. Interestingly, the transfer of gut microbiota from RYGB-treated mice to germ-free mice led to weight loss in both germ-free mice as well as sham-operated mice, with germ-free mice exhibiting a greater decrease in fat mass than sham-surgery counterparts [10]. It is also important to note that many of the microbial changes occur distant from the area of surgical manipulation, typically observed in the distal gut [10].

Moreover, while the aforementioned study [36] reported greater alpha-diversity in SG patients, the amount of weight gain following RYGB surgery in obese individuals was shown to be associated with the degree of alpha-diversity [40]. For example, those exhibiting no recurrent weight gain five years after surgery had a significantly higher alpha-diversity, when compared to the RYGB with recurrent weight gain [40]. The non-regain group also exhibited increased *Akkermansia*, a gut microbial genus well-known for its beneficial effects in maintaining gut barrier integrity [41]. It is worth noting that over a five-year period, several factors such as environment, diet, and medication can influence changes in the gut microbiota. These alterations are not directly linked to the surgical procedure itself. However, the study evaluating the role of alpha-diversity in recurrent post-surgical weight gain [40] is important in showing the role of gut microbes in weight maintenance following surgery as well as changes directly following the procedure even though external influences on microbiota are difficult to control.

Comparative studies in humans have shown similar increases in the aero-tolerant *Escherichia* and *Streptococcus* genera following RYGB, but have also consistently described conserved increases in *Veillonella* spp. with notable decreases in *Blautia* spp. [28,30,42,43] (Figure 1). These specific changes are thought to be in part due to lower gastric acid exposure allowing native oral cavity bacteria to prevail in greater abundances [43]. Interestingly, *Veillonella* has been shown to exert bile acid-sensitive properties, which may enable the suppression of toxic bile acid synthesis [44]. Importantly, these perturbations in the production of bile acids, as well as other gut microbial metabolites including trimethylamine-N-oxide (TMA-O), neurotransmitter precursors, and branched chained amino acids all play important roles in promoting weight loss following RYGB [45]. These changes were more pronounced following RYGB when compared to SG [45], and the effects of these metabolites will be further discussed in a later subsection.

### 2.2. Sleeve Gastrectomy (SG) and Gut Microbiota

**Figure 2:**
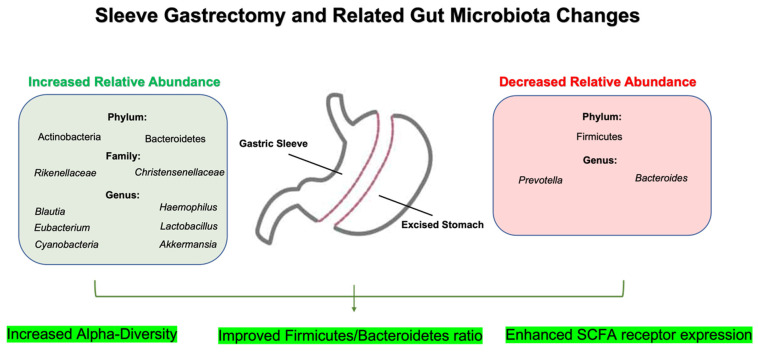
Sleeve Gastrectomy (SG) and Related Gut Microbiota Changes. The middle graph depicts the SG procedure. Approximately 70–80% of the greater curvature is excised, creating a gastric sleeve. Following SG, relative abundance of phyla Actinobacteria and Bacteroidetes are increased, while Firmicutes is decreased. Family *Rikenellaceae* and *Christensenellaceae* are increased. Genera *Blautia*, *Akkermansia*, *Eubacterium*, *Lactobacillus*, *Cyanobacteria*, and *Haemophilus* are characteristically increased, while *Blautia* is decreased. Overall, RYGB increases alpha-diversity, improves the *Firmicutes*/*Bacteroidetes* ratio, and enhances SCFA receptor expression. Abbreviations: SCFA, short-chain fatty acids.

Sleeve Gastrectomy (SG), also termed vertical sleeve gastrectomy (VSG), is a procedure primarily conducted laparoscopically, in which up to 80% of the greater curvature of the stomach tissue is removed [46]. SG mainly promotes weight loss through the mechanical restriction of food intake, but it also affects the levels of satiety peptides, thereby influencing changes in energy balance [47]. Like RYGB, the comprehensive impact on weight loss can also be partially explained by taxonomic changes in the gut microbiota [9]. As the procedure differs from RYGB, so do certain changes in gut microbial composition following surgical intervention. For example, recent studies have shown that *Blautia* was characteristic in SG, along with other changes including relatively increased abundances of *Blautia*, *Eubacterium*, and *Haemophilus* [30], while *Veillonella* was associated with RYGB [42]. Contrary to RYGB, in a large cohort of obese individuals who underwent SG, *Bacteroides fragilis* was shown to be decreased along with *Prevotella*, though similar improvements in the *Firmicutes*/*Bacteroidetes* ratio were noted after 3 months and 12 months [48] (Figure 2). At 12 months, the phylum Actinobacteria was also increased [48]. Interestingly, in one study [49], the abundance of *Bacteroides fragilis* has been shown to have positive associations with estimated weight loss in the SG group, while a negative correlation was found between RYGB patients and body mass index up to six months after surgery. Nonetheless, the literature does not clearly describe how *Bacteroides fragilis* aids in weight loss following bariatric surgery. Research indicates that supplementing this bacterium may actually worsen metabolic markers in both obese individuals and animal models [50,51]. However, it should be noted that these studies were not conducted in subjects who underwent bariatric surgery.

In addition to these distinct gut microbial changes, SG promotes favorable changes in gut metabolites. In a rodent model of SG, it was shown that fecal SCFA concentrations increased along with an up-regulation of its receptor, G-protein receptor 43 (GPR43) [52]. Importantly, the marked changes in fecal SCFA post-SG are shown to be positively correlated with metabolic parameters including serum triglycerides and total cholesterol [53]. These changes observed in SG can be partially explained by relative abundances in SCFA-producing bacteria including *Blautia* and *Eubacterium*, as previously discussed, as well as *Lactobacillus* and *Bifidobacterium*, which were elevated following surgery [48,54,55]. Furthermore, *Akkermansia* abundance is increased following both RYGB and SG [28,30,36], which promote the growth of SCFA-producing bacterial genera and reduce species associated with inflammatory processes [56] (Figure 2). In summary, the literature pertaining to gut microbial changes following specific bariatric procedures is rapidly evolving; however, the results in humans are still inconsistent across studies considering that numerous environmental factors can influence the variability in outcomes.

## 3. Bariatric Surgery, the Microbiota–Gut–Brain Axis, and Peripheral Targets

As previously described, bariatric surgery induces significant changes in microbial composition, which influences signaling via the gut–brain axis. The mechanisms by which this occurs include bidirectional communication between the microbiota–gut–brain axis through the vagus nerve via local and systemic inflammation, as well as the production of microbiota metabolites including SCFA and bile acids.

### 3.1. Bariatric Surgery, Gut Microbiota, and Vagal Contribution

**Figure 3:**
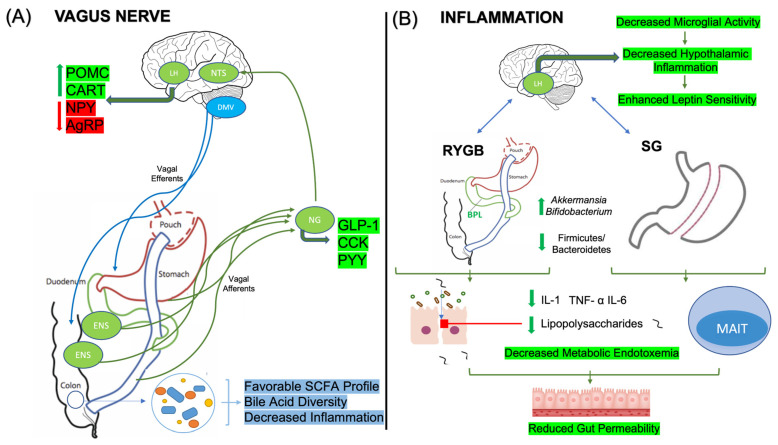
(**A**,**B**) Microbiota–Gut–Brain Axis following RYGB and SG. (**A**) Vagus Nerve. Sensory information from vagal afferents in the ENS is conveyed to the nodose ganglion. RYGB-induced changes in microbiota lead to favorable SCFA profile, increased bile acid diversity, and decreased inflammation. RYGB alters neuronal activity enhancing satiety signaling through GLP-1, CCK, and PYY. The NG further transmits signals relayed from the gut to the NTS and lateral hypothalamus. At the level of the lateral hypothalamus, POMC, cocaine-, and CART are upregulated while NPY and AgRP are down-regulated following RYGB. Vagal efferents from the dorsal motor nucleus of vagus relay brain signals to the gut to influence gut peptides. (**B**) Inflammation. Microbiota-related changes including increases in *Akkermansia* and *Bifidobacterium*, as well as overall improvements in the *Firmicutes*/*Bacteroidetes* ratio mitigate inflammatory processes in the periphery and brain. In the brain, hypothalamic inflammation is attenuated due to decreased microglial activity, contributing to improved leptin sensitivity. In the periphery, release of inflammatory cytokines and serum lipopolysaccharides are decreased, thereby decreasing metabolic endotoxemia. In SG, mucosa-associated invariant T-cell expression is enhanced in colonic tissue. These changes collectively improve gut barrier integrity and reduce gut leakage. Abbreviations: POMC, proopiomelanocortin; CART, cocaine-, and amphetamine-regulated transcript; NPY, neuropeptide Y; AgRP, agouti-related peptide; LH, lateral hypothalamus; NTS, nucleus tractus solitarius; DMV, dorsal motor nucleus of vagus; NG, nodose ganglion; GLP-1, glucagon-like peptide 1; CCK, cholecystokinin; PYY, peptide YY; ENS, enteric nervous system; SCFA, short-chain fatty acids; RYGB, Roux-en-Y gastric bypass; SG, sleeve gastrectomy; BPL, Biliary-Pancreatic Limb; IL-1, interleukin-1; TNF-α, tumor necrosis factor alpha; IL-6, interleukin-6; MAIT, mucosa-associated invariant T-cell.

The vagus nerve is the longest cranial nerve and provides the widespread innervation of many major organ systems, as well as muscles and fat depots. The vagus is a mixed nerve composed of roughly 80% afferent and 20% efferent fibers, providing a critical interface between the brain and periphery, serving as a key point for interoception as well as integration of central and autonomic nervous systems [57]. Thus, the vagus nerve and afferent vagal nerve fibers innervating the gastrointestinal tract serve an important role in weight loss following bariatric surgery. For example, the vagotomy of the celiac branch in addition to RYGB significantly modulated weight loss in rodents in the first 40 days post-surgical intervention [58]. By contrast, animals that underwent RYGB without vagotomy lost significantly more weight and suppressed food intake, indicating an integral role of vagal afferents in these processes [58]. The behavioral data was corroborated by neuronal trace studies showing a significant reduction in the tracer-labeled neurons in the nodose ganglia (NG) and dorsal motor nucleus of vagus (DMV) following RYGB, but not SG. In addition, SG intervention enhanced the density of vagal afferents in the nucleus tractus solitarius (NTS), while RYGB-treated rats exhibited the opposite effect [59] (Figure 3A). Similarly, it was found that only the RYGB-activated microglia in these vagal structures influence vagus nerve-mediated changes in gut–brain communication. These findings suggest that of the two procedures, RYGB inherently damages vagal innervation to a greater extent than SG; however, both procedures influence vagal communication to an extent.

Gut microbiota and their metabolites have been shown to influence vagal afferent firing frequency as well as activate vagal sensory neurons in the enteric nervous system (ENS) to promote variable effects on satiety and behavior [21]. Indeed, satiety-mediated hormone receptors, including those for ghrelin, leptin, GLP-1, PYY, and CCK, are in proximity with vagal afferent fibers [21,60], communicating changes in brain chemistry to promote satiation (Figure 3A). For example, the introduction of intraluminal *Lactobacillus* spp. to the jejunum of Swiss Webster mice augmented vagal afferent firing in the mesenteric nerve, reduced anxiety-related behavioral scores, and exhibited effects that were absent in vagotomized mice [61]. Furthermore, the vagus nerve and neurons within the ENS express toll-like receptors, particularly at the level of the NG, which are influenced significantly by the production of lipopolysaccharides (LPS) by enteric bacteria [62]. In turn, negative changes towards more inflammatory gut profiles can negatively mediate satiation and impair leptin signaling [63]. In addition to mediating satiation signals, the vagus nerve serves as an important player in neurotransmitter biosynthesis, notably dopamine and serotonin. As such, the inoculation of dysbiotic bacteria increased neuronal activation within the NTS and the hippocampus and decreased neurotransmitter biosynthesis [64]. Again, these effects on neurogenesis, neuroinflammation, and biosynthesis required an intact vagus nerve and were not seen in previously vagotomized animals. Similarly, neurotransmitter precursors produced by intestinal bacteria activate enteroendocrine cells through the binding of the transient receptor potential ankyrin A1 (TRPA1), which acts on enteric and vagal nerves. Of note, *Edwardsiella tarda* is found to promote signaling through this pathway as well as contribute to serotonin secretion in the intestine [65]. Taken together, the evidence supports the role of gut bacteria influencing satiety peptides and neurotransmitters that regulate reward and feeding pathways, all of which can be modified by bariatric surgery.

Although the vagus nerve is crucial in these mechanisms, certain microbiota-driven effects on weight loss post-bariatric surgery occur independently of the vagus nerve’s participation. In particular, the genus *Parabacteroides* has been correlated with positive effects in post-surgical mice [66]. In a recent study, mice undergoing SG with truncal vagotomy had increased *Eubacterium*, *Prevotella*, and *Parabacteroides* that were correlated with enhanced weight loss, though *Parabacteroides* was exclusive to the subgroup that underwent SG with truncal vagotomy, which experienced greater weight loss compared to both the sham group and the group that had only SG [66]. *Parabacteroides* is known for its anti-obesity effects, although these benefits are primarily immunomodulatory and occur through the enhancement of the gut barrier [67]. In addition, *Parabacteroides* and *Akkermansia* have been shown to activate the leptin signaling pathway to increase brown adipose tissue thermogenesis, which promotes energy expenditure [68]. This underscores the multi-modal mechanisms by which microbiota contribute to weight loss following bariatric surgery both with or without vagal contribution.

### 3.2. Bariatric Surgery, Gut Microbiota, Cytokines, and Inflammation

Bariatric surgery significantly alters the inflammatory profile of the gut, including related cytokines and intestinal barrier integrity through the modulation of gut microbiota. For example, in obese rodent models following RYGB intervention, improvements in LPS concentrations as well as reductions in pro-inflammatory cytokines, interleukin-1 (IL-1), interleukin-6 (IL-6), and tumor necrosis alpha (TNF-α) were observed in parallel with beneficial changes in cecal microbiota, including enhanced amounts of *Bifidobacterium* and *Veillonella* [69]. Further, LPS, when bound to its receptor, TLR-4, in excess, serves as a major driver of metabolic endotoxemia and intestinal barrier permeability by down-regulating the tight junction proteins zonulin-1, claudin, and occludin [70]. As a result, chronically elevated states of LPS allow for its leakage into the bloodstream, and it promotes systemic inflammation through the enhanced release of pro-inflammatory cytokines [71]. Bariatric surgery has been shown to counteract these adverse effects, with RYGB leading to an increase in microbiota metabolites that help mitigate endotoxemia [72] (Figure 3B).

Specifically, SCFAs were increased in association with *Akkermansia muciniphila* over a 6-month post-operative period in obese patients with a concomitant decrease in lipopolysaccharide-binding protein, contributing to weight loss and improved insulin resistance. Post-surgery, the release of cytokines is reduced via suppression of the NLRP3 inflammasome signaling pathway [73]. This inflammasome is a critical constituent of the innate immune system and is activated through a plethora of stimuli including reactive oxygen species, ionic flux, overgrowth of unfavorable microbes, and mitochondrial dysfunction, all of which are influenced by the dysbiosis of gut microbes [74]. As such, duodenal–jejunal bypass, a less common subtype of bariatric surgery, has been shown to alleviate infiltrating macrophages to enhance glucose tolerance and weight loss while improving β-cell function, an important marker of type 2 diabetes mellitus.

Patients undergoing SG have also been shown to have similar augmentations in immunomodulation following the procedure. Evidence shows improvements in cytokine profiles, specifically with significant reductions in IL-1β and TNF-α in conjunction with an improvement in the *Firmicutes*/*Bacteroidetes* ratio [75] (Figure 3B). Interestingly, these findings were accompanied by improved cognitive function with alterations in cytokines such as interleukin-4 (IL-4), IL-1β, and TNF-α that correlated with cognitive enhancements. A decrease in Firmicutes was positively correlated with TNF-α, while increased Bacteroidetes was negatively correlated with IL-4 [75]. In addition to cytokine-mediated effects, SG also induces changes in adaptive immunity via the modulation of regulatory T cells (Tregs) up to 12 months post-operation [76]. Specifically, the frequency of mucosal-associated invariant T (MAIT) cells was enhanced in the colonic mucosa, a subset of T-cells that promote intestinal mucosal integrity and protection against pathogenic bacteria [77]. Simultaneously, SG led to an increase in alpha-diversity over the next year, up until the conclusion of the study, and was associated with a substantial total weight loss of 30% [76].

The inflammatory benefits conferred following bariatric surgery are not limited to the gut alone but also extend to improving neuroinflammation through microbiota–gut–brain crosstalk. In recent years, hypothalamic and local neural inflammation has been an area of interest in patients undergoing RYGB due to its importance in regulating satiation signals and the pathogenesis of obesity [78,79]. Leptin resistance is, to some extent, secondary to hypothalamic inflammation, with neuroinflammatory mediators such as excessive microglial and astrocyte activation contributing to its onset [80,81]. The microbiota and its metabolites, such as SCFAs, play a crucial role in the maturation of microglia, the brain’s primary macrophages. Studies in SCFA-receptor knockout mice have demonstrated that these animals exhibit dysfunctional and immature microglia [82]. In addition to leptin, orexigenic neuropeptides are also negatively affected in the setting of hypothalamic inflammation with NPY/AgRP co-expressing neurons through gut–brain crosstalk with microglia being heavily involved [83]. Recent studies have suggested that hypothalamic AgRP neurons may also reciprocally promote TNF-α release through a top-down mechanism to worsen endotoxemia and energy balance [84]. Previous work suggests that bariatric surgery mitigates hypothalamic inflammation via gut–microglia crosstalk through a weight loss-independent manner [78] (Figure 3B). It was found that RYGB alleviated hypothalamic TLR4 signaling, decreased the plasma lipopolysaccharide-binding protein, and lowered the expression of microglia and endoplasmic reticulum stress to improve leptin sensitivity [78]. Microbiota were the main players in this study, as antibiotic introduction following RYGB worsened the anorexigenic action of leptin. In another study, similar outcomes were noted, with RYGB normalizing gene expression and being linked to inflammation in the hypothalamus, reducing excessive microglial activity within only eight weeks in rodent models [85]. Importantly, satiety hormones and neuropeptides, leptin and AgRP, respectively, were also normalized at the same time. In summary, fluctuations in gut microbiota following bariatric procedures significantly influence local and systemic inflammation to alter peripheral and neural mediators controlling food intake.

### 3.3. Bariatric Surgery, Gut Microbiota, Nutrient-Sensing Receptors, and SCFA

SCFAs, most notably butyrate, propionate, and acetate, are metabolic byproducts of gut microbiota, resulting from the fermentation of dietary carbohydrates and resistant starches that are otherwise indigestible to humans [86]. The concentration of these SCFAs is dependent on multiple factors, including, but not limited to, relative abundances of SCFA-producing bacterial genera, carbohydrate or starch intake, states of systemic inflammation, and pathogenic microbial overgrowth [87]. Their benefits are multifaceted, with improvements in multiple metabolic and obesity-related parameters such as satiety peptide regulation, reductions in gut barrier permeability, body weight reduction, improved insulin sensitivity, and mitigated low-grade systemic inflammation [88,89,90,91]. For example, propionate infusion in animal models promotes the increased release of PYY and GLP-1 from colonic tissue, while down-regulating hypothalamic orexigenic AgRP expression and up-regulating CART, an anorexigenic neuropeptide [91]. SCFAs exhibit weight loss-related effects through binding to their nutrient-sensing receptors GPR41 and GPR43, also known as free fatty acid receptor 2/3 (FFAR2/FFAR3), which are expressed in adipocytes and colonic epithelial cells [92]. These nutrient-sensing receptors are integral in processes related to energy balance, as GPR43-deficient mice fed a normal diet become obese, while those overexpressing GPR-43 do not gain weight even when on a high-fat diet, presumably through the GPR-43-mediated inhibition of fat accumulation and suppressed insulin signaling [93].

**Figure 4:**
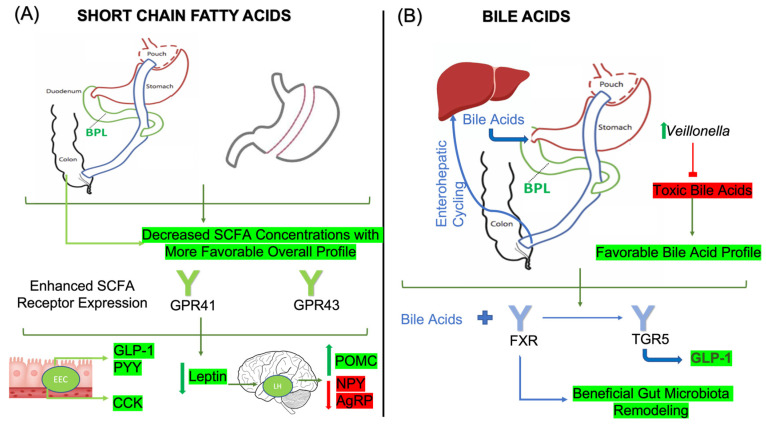
Microbiota–Gut–Brain Axis following RYGB and SG. (**A**) Short-Chain Fatty Acids. Both procedures reduce overall SCFA concentrations due to nutrient intake restriction; however, overall, SCFA profile is more favorable. Expression of nutrient-sensing receptors, GPR41 and GPR43, is enhanced in colonic mucosa following bariatric surgical procedures. Activation of nutrient-sensing receptor causes release of GLP-1, PYY, and CCK from enteroendocrine cell. It also influences leptin activity by relaying signals to the brain to alter POMC, NPY, and AgRP neuronal expression. These changes contribute to the feeling of satiety following bariatric surgery. (**B**) Bile Acids. Primary bile acids are made in the liver and released into the biliary-pancreatic limb in Roux-en-Y gastric bypass. Roux-en-Y reduces the distance to the terminal ileum where bile acids undergo enterohepatic cycling to be further processed and re-released into the intestinal tract. At the same time, favorable changes in gut microbiota profile following the procedure, such as relative increases in bile acid-sensitive *Veillonella*, further suppresses toxic bile acid synthesis, promoting an overall more favorable bile acid profile. Bile acids bind to Farsenoid X Receptor and Takeda G protein-coupled receptor 5 to influence satiety peptide expression. Farsenoid X Receptor activity can further activate Takeda G protein-coupled receptor 5 as well as reciprocally remodeling gut microbiota. Takeda G protein-coupled receptor 5 increases GLP-1 release. Abbreviations: BPL, biliary-pancreatic limb; SCFA, short-chain fatty acids; GPR41, G-protein coupled receptor 41; GPR43, G-protein coupled receptor 43; EEC, enteroendocrine cells; GLP-1, glucagon-like peptide 1; CCK, cholecystokinin; PYY, peptide YY; LH, lateral hypothalamus; POMC, proopiomelanocortin; NPY, neuropeptide Y; AgRP, agouti-related peptide; FXR, Farsenoid X receptor; TGR5, Takeda G protein-coupled receptor 5; RYGB, Roux-en-Y gastric bypass.

Bariatric surgery has been shown to significantly alter both short-chain fatty acids and the expression of nutrient-sensing receptors post-procedure, although some of the findings are somewhat inconsistent [94,95]. Due to the inherent procedural goal of reducing caloric intake through mechanical restriction and/or nutrient malabsorption in RYGB, it would make sense that SCFA may be unchanged or reduced following surgery given the overall reduction in carbohydrate and starch substrates available for gut microbiota fermentation [96]. A study of 90 individuals having undergone either RYGB or SG showed decreased fecal SCFA concentrations of the common SCFAs, while their branched counterparts, isobutyric, isovaleric, and isocaproic acid, were increased [94]. This suggests a proteolytic pattern of fermentation due to low carbohydrate intake, which can be counteracted via an appropriate diet. The study subjects generally reduced food intake, except for protein, of which the overall intake was increased, possibly accounting for the observed results [94]. These findings are in line with a recent study indicating a 27% reduction in SCFAs after bariatric surgery. The study suggests that enhancing the consumption of food groups such as dairy, fruits, vegetables, nuts, and grains restores SCFA levels, as they play important roles in enhancing weight loss [97] (Figure 4A). By contrast, a similar observational study involving 51 patients undergoing RYGB exhibited increased butyrate and propionate, with decreased acetate at 12 months post-surgery. Similarly, this study also demonstrated an increase in isobutyrate and isovalerate acids [95]. Elevations in butyrate, propionate, and isobutyrate were noted to be negatively correlated with BMI, indicating a beneficial role in promoting weight loss post-surgery, while isobutyrate also increased insulin sensitivity scores.

In animal models, the data generally point to a relative increase in the abundance of SCFAs and related receptors [54,98]. For example, rodent models undergoing three different types of bariatric surgery, including RYGB, SG, and biliopancreatic diversion with a duodenal switch, had a more favorable SCFA profile that was associated with positive correlations in PYY levels [98]. Similarly, obese rats undergoing SG had higher acetate and butyrate concentrations in plasma and stool, with related increases in GPR43 mRNA expression in the ileum and epididymal fat [54] (Figure 4A). These findings were accompanied by the presence of *Lactobacillus* and *Lactococcus*, which are a notable SCFA-producing bacterial genera. However, it should be noted that, in these studies, the dietary intake of rats can be monitored more closely, while those in human studies are generally self-reported and not well monitored. As previously mentioned, dietary content plays a significant role in SCFA concentrations; therefore, these changes may not be directly related to surgical intervention but are rather secondary to changes in total dietary intake and food preference. Nevertheless, the presence of SCFAs promotes beneficial effects on metabolic parameters and energy balance.

### 3.4. Bariatric Surgery, Gut Microbiota, and Bile Acids

Bariatric surgery significantly alters the enterohepatic cycling of bile acids and bile acid composition via gut microbiota-dependent mechanisms to further contribute to post-procedural weight loss and satiety [99,100]. Primary bile acids are synthesized in the liver and can be chemically transformed by gut microbial enzymes to produce secondary bile acids via hydroxylation and dihydroxylation reactions [101]. Further, bile acids that have been conjugated such as ursodeoxycholic acid (UCDA) and taurodeoxycholic acids (TCDA) may also be deconjugated by microbiota bile salt hydrolase activity to free conjugates like taurine, which can reciprocally aid in favorably remodeling gut microbial composition [99,101,102]. These enzymatic processes serve to detoxify these substrates and promote bile acid diversity [99].

Importantly, bile acids contribute to metabolic, endocrine, and hormonal function through interactions with their receptors, the Farsenoid X Receptor (FXR) and Takeda G protein-coupled receptor 5 (TGR5) [103]. These receptors serve as crucial modulators of weight loss and metabolic benefits as studies have demonstrated that both FXR-deficient and TGR5-deficient rodent models exhibit lesser weight loss and worse insulin resistance as compared to their wild-type counterparts after bariatric surgery [104,105]. Gut microbiota-mediated increases in bile acid diversity directly influence receptor activity [106], as primary bile acids are more potent stimulators of FXR, while secondary bile acids and specifically taurine-conjugated bile acids bind with a stronger affinity to TGR5 [107,108]. As such, recent studies have demonstrated that gut microbiota are an important player in stimulating the GLP-1 response through TGR5 signaling [109] (Figure 4B). Similarly, FXR agonism confers beneficial changes in gut microbiota composition, leading to the activation of TGR5, stimulation of GLP-1 release, and formation of brown adipose tissue, which is a significant indicator of net energy expenditure [110]. These findings also have implications for reward pathways and food-driven behaviors. TGR5 knockout mice display reduced behaviors associated with reward, whereas the restoration of TGR5, specifically in the nucleus accumbens, reverses these deficits following bariatric surgery [111].

Following bariatric surgery, bile acid diversity is notably increased, an effect with important implications in post-surgical weight loss independent of caloric restriction [45,112,113,114]. Researchers have identified changes in *Veillonella* and *Blautia* that are implicated in these mechanisms, with *Veillonella* typically increasing and *Blautia* decreasing following RYGB [113]. *Veillonella* is a bile acid-sensitive bacterium thought to mediate its beneficial effects by suppressing the synthesis of toxic bile acids [44]. This is demonstrated through the introduction of an FGF19 analog, which is expressed after FXR binding to promote a dose-dependent enrichment in *Veillonella*, which, in turn, promotes beneficial changes in bile acids [44] (Figure 4B). Interestingly, following RYGB, bile acid concentrations as well as FGF19 are increased, an effect that is not observed after medical treatment [115]. While recent findings support the increase in bile acid in RYGB patients, the extent to which they are increased in SG is less significant. For example, individuals undergoing SG may achieve reduced bile acid excretion through increased bacteria alpha-diversity, particularly in associated taxa including *Rikenellaceae* and *Christensenellaceae* [114]. However, a recent meta-analysis has shown that although bile acid concentrations can be increased in SG patients, the difference pre- to post-surgery in these subgroups was not significant [116]. These differential findings can be attributed in part to the decreased distance following RYGB required for bile acids to reach the terminal ileum, where the enterohepatic salvaging of bile acids occurs [117]. Therefore, bile acid pools can be regarded as both surgery-type dependent as well as related perturbations in gut microbiota following surgical intervention.

## 4. Bariatric Surgery, Gut Microbiota, and Regulatory Pathways Controlling Food Intake

Bariatric surgery confers physiological changes to gut peptides and neuropeptides involved in the sensations of hunger and satiation [118]. Pre-prandial hunger is initiated through the activity of orexigenic gut hormones such as ghrelin and neuropeptides including NPY and AgRP, as well as through hedonic control via the reward pathways [119]. Satiation, on the other hand, occurs post-prandially; after-meal contents promote gastric distension, stimulating stretch receptors and transmitting signals through the vagus nerve to alter brain chemistry and promote the release of anorexigenic hormones and neuropeptides including leptin, POMC, and CART [119]. Furthermore, enteroendocrine signaling enables the local detection of nutrients, which activates vagal afferents in the ENS, triggering the release of gut hormones such as CCK, GLP-1, and PYY [120]. The gut microbiota and their nutrient-sensing receptors are intricately implicated in these processes, as it has been shown that germ-free mice remain leaner following the consumption of higher amounts of calories when compared to their conventionally raised counterparts [121]. Similarly, the depletion of microbiota promotes the formation of brown adipose tissue while concurrently reducing the inhibition of leptin activity [122]. We have also recently shown that these effects may be secondary to the effects of satiety hormones and neuropeptides [123]. Additionally, exogenous leptin administration in these same GF mice improved GLP-1, PYY, and ghrelin concentrations, which were all decreased at baseline compared to conventionally raised counterparts [123]. Further, reward pathways are also affected, as gut microbial composition changes. For example, an increased *Prevotella*/*Bacteroides* ratio is linked with obesity, as well as with enhanced activity of nucleus accumbens, a subcortical brain structure central to pleasure, reward, and food addiction [124]. Bariatric surgery, as previously mentioned, alters these processes, including vagal signaling as well as the relative abundances of certain gut microbiota, thereby relaying generally positive effects on neuropeptides and satiety hormones through microbiota–gut–brain communication. The mechanistic contribution of gut microbiota as it pertains to post-bariatric surgical changes in hunger, satiety, and reward will be described in further detail in the following subsections.

### 4.1. Bariatric Surgery, Gut Microbiota, and Effects on Ghrelin and Leptin

Leptin and ghrelin are anorexigenic and orexigenic hormones, respectively, that contribute significantly to energy balance and related processes [125]. Leptin and ghrelin receptors are present throughout the CNS, most notably in the arcuate nucleus (ARC) of the hypothalamus. The activation of their receptors influences the expression of orexigenic and anorexigenic neuropeptides in a reciprocal fashion, with ghrelin promoting NPY and AgRP neuronal signaling while leptin attenuates signaling from neurons co-expressing these neuropeptides [126,127]. Ghrelin is a gut hormone released after fasting and associated hunger [125]. However, states of hyperleptinemia and related leptin resistance are characteristic of long-standing obesity, due to the direct proportion of the amount of adipose tissue and circulating leptin concentrations [128]. Nevertheless, both the ghrelinergic and leptin systems are significantly influenced by the presence of microbiota as well as its composition. For example, microbiota depletion promotes the browning of adipose tissue, thereby reducing leptin levels and improving leptin resistance [122]. Conversely, conventionally raised mice with intact gut microbiota exhibit a diminished response to exogenous leptin, indicating a component of leptin resistance when compared to their germ-free counterparts [129]. An inhibitor of leptin signaling, suppressor of cytokine signaling 3 (SOCS3), is also elevated in conventionally raised mice, indicating a state of hyperleptinemia as SOCS3 expression is induced in response to circulating leptin levels [129]. Similarly, the transition to a more inflammatory microbial composition induced by a high-fat dietary intervention induced the hypermethylation of the leptin promoter, conferring resistance [130]. Further, the introduction of *Lactobacillus rhamnosus* may improve leptin sensitivity in a diet-induced model of obese rodents [131]. Conversely, ghrelin is negatively correlated with *Prevotella* and *Bacteroides*, while it is positively correlated with beneficial SCFA-producing bacterial genera such as *Bifidobacterium*, *Lactobacillus*, and *Eubacterium* [132]. Taken together, these findings provide strong evidence for the role of gut microbiota in influencing leptin and ghrelin activity.

Bariatric surgery significantly alters ghrelinergic signaling and alleviates leptin resistance, both dependent and independent of its effects on microbiota composition, with post-surgical reductions seen in both serum ghrelin and leptin concentrations [133,134] (Table 1). These processes are complex and multifactorial as ghrelin levels decrease in SG and increase in RYGB, at least when measured directly after surgery, although serum ghrelin is decreased at 1 year following both bariatric surgical subtypes [135]. In RYGB, vagal afferents are shown to contribute to these processes as vagal nerve denervation hampered the extent of these benefits in short-term responses to meal ingestion [136]. As previously mentioned, the NG, which harbors vagal afferents from the gut, is affected by alterations in gut microbiota, particularly through processes that promote inflammation. Following RYGB, alterations in *Escherichia coli* and *Bacteroides* abundance correlate with serum leptin and fat mass [137]. Specifically, an increase in *Bacteroides* is associated with lower serum leptin and associated decreases in fat mass [137] (Table 1). As mentioned, *Bacteroides* concentrations are notably increased following bariatric surgery, potentially having a role in mitigating leptin resistance.

Further, bariatric surgery attenuates inflammatory processes through the remodeling of gut microbiota, which consequently improve the signaling of satiety hormones, including leptin [162].

In states of obesity, the gram-positive bacterial production of lipoteichoic acid (LTA) or its gram-negative equivalent, LPS, induces local inflammation, diminishing NG sensitivity to leptin, but also to CCK, GLP-1, and PYY [162]. RYGB also mitigates TLR4 signaling to restore anorexigenic actions of leptin as a direct result of changes in the gut microbiota [78]. Therefore, improvements in microbiota composition may confer improved hormone sensitivity within the ENS to influence feelings of satiety and the improvement of hyperleptinemia-induced obesity. SG, on the other hand, confers benefits in leptin sensitivity through other microbiota-dependent mechanisms [52] that also involve improvement in peripheral inflammatory processes. For example, 10 weeks post-operation, rodent models that underwent SG exhibited reduced leptin levels in conjunction with an upregulation of SCFA and expression of colonic GPR43 [52]. In addition to the microbial effects on local inflammation, hypothalamic inflammation is a significant contributor to leptin resistance [163], with notable improvements following bariatric surgery [85]. Improvements in hypothalamic inflammation in a time-dependent fashion following surgical intervention are thought to result from improvements in peripheral inflammation [138] and may result from microbiota-related processes [78]. Further, the beneficial microbiota-related changes are shown to improve hypothalamic gliosis and inflammatory signaling, contributing to enhanced leptin responsiveness, specifically through reductions in SOCS3 [78]. In the same study, inducing a state of dysbiosis diminished these parameters, suppressing leptin’s effect on satiety.

The mechanisms of ghrelin–gut microbiota interplay are not as well defined; however, microbial-derived metabolites including SCFAs, LPS, and hydrogen sulfide (H2S) may directly or indirectly modulate ghrelin secretion from enteroendocrine cells [164]. Interestingly, *Faecalibacterium*, which is increased post-RYGB, is negatively correlated with ghrelin concentrations [137]. *Faecalibacterium* is an SCFA-producing bacterial genera thought to exert its benefits through the production of this neuroactive metabolite, as SCFAs bind to GPR43 to promote down-stream inhibitory effects on ghrelin secretion [165,166]. Ghrelin exerts orexigenic effects through binding with its receptor, the growth hormone secretagogue receptor (GHSR1a), which is expressed throughout the central and peripheral nervous systems [167]. Therefore, the antagonist effects observed by Furet et al. were shown to target the GHSR1a receptor, with decreased ghrelin-induced calcium release and receptor internalization noted following butyrate treatment [168].

Further, metabolic endotoxemia and LPS produced by gastric and enteric bacterial species may influence signaling via GHSR1a [139]. It was shown that *H. pylori*-mediated LPS release promoted the convergence of GHR1a and TLR-4 signaling pathways, indicating that LPS can modulate ghrelin release through TLR4-activating mechanisms [139]. Lastly, the production of H2S through gut microbiota paradoxically suppresses ghrelin secretion [140]. Specifically, the introduction of H2S donor molecules in rodents attenuated ghrelin release through the activation of the protein kinase B, a down-stream signaling molecule in the GHSR1a signaling pathway. Not only did H2S suppress ghrelin signaling, but it also prolonged the post-prandial drop of serum ghrelin for 4 h while concurrently reducing food intake in these mice [140]. Notably, *Escherichia*, *Veillonella*, *Clostridium*, *Bacteroides*, and *Streptococcus*, all of which are characteristically elevated following RYGB, contain an H2S-producing capability, indicating a significant role in post-surgical microbial changes as a contributor to reduced ghrelinergic signaling and serum hormone concentrations. Importantly, GHSR1a is also implicated in the hedonic control of food intake; however, this will be further discussed in a later subsection.

### 4.2. Bariatric Surgery, CCK, and Gut Microbiota

Cholecystokinin (CCK) is a satiety hormone produced by duodenal I-cells causing a delay in gastric emptying through vago-vagal reflexes to reduce food intake [169]. CCK is secreted in response to luminal stimuli including microbiota metabolites such as amino acids, fatty acids, and endocannabinoids [170]. CCK binds to its receptors, CCK-1, located mainly in the gut, and CCK-2, located mainly in the brain, to influence satiation signaling [171]. Substantial evidence shows that following bariatric surgery, serum CCK levels are increased post-prandially [141,172] (Table 1), although the direct influence of gut microbiota is not as well understood. One proposed mechanism is through microbiota’s effects on circulating endocannabinoids and related receptors, notably the cannabinoid 1 receptor (CB1R), as these receptors also reside in intestinal I-cells [173]. Endocannabinoids are naturally occurring neurotransmitters that are shown to influence peripheral and neural pathways controlling food intake [174], through the alteration of vagal afferent sensitivity within the gut as well as the reduction in synaptic plasticity and neurogenesis associated with hyperphagia [175,176]. Further, in diet-induced obesity, it has been shown that small intestinal signaling via the CB1R pathway inhibits nutrient-mediated CCK release via gut–brain dependent mechanisms [177]. For example, diets shown to have inflammatory effects induce unfavorable changes in the gut microbiota and elevate endocannabinoid tone, further hampering CCK release [170]. This is supported by evidence showing a positive correlation between endocannabinoids and LPS levels [178]. Importantly, bariatric surgery reduces circulating levels of endocannabinoids ligands, namely 2-arachidonoylglycerol (2-AG), anandamide (AEA), and oleoylethanolamine (OEA), with direct associations with clinical benefits [179,180]. More specifically, sustained weight loss following RYGB was significantly correlated with reduced endocannabinoids and related increases in CCK [142]. These alterations in post-surgical endocannabinoid levels also correlated with reduced leptin levels as well [142]. In mice, these studies have been further replicated using a CB1R antagonist mimicking effects on weight loss. This resulted in a shift in the production of brown adipose tissue and increased splanchnic nerve activity as seen in RYGB, all of which were attenuated by AEA supplementation [181]. In addition to endocannabinoids, SCFAs also influence CCK receptor signaling through vagus-dependent pathways [143]. GPR41 expression in the NG has a critical role, as vagal-GPR41-deficient mice increased food intake and hampered CCK receptor signaling [143]. Taken together, these studies support the influential role of microbiota metabolites including the endocannabinoid system in promoting weight loss in part through altering CCK secretion following bariatric surgery.

### 4.3. Bariatric Surgery, Gut Microbiota, GLP-1, and GLP-2

GLP-1 and GLP-2 are gut hormones that are enzymatically cleaved from proglucagon and are secreted by intestinal L-cells [182]. Of the two, GLP-1 has been shown to be more associated with regulating body weight through a myriad of mechanisms including slowing the rate of gastric emptying, promoting weight loss, and improving glucose metabolism [183,184]. Following bariatric surgery, the serum level of GLP-1 is significantly increased in as early as one week post-intervention [144] (Table 1). These increased levels were present even in a 10-year follow-up study, demonstrating that even with recurrent weight gain, GLP-1 levels remained elevated [145]. This led to the conclusion that satiety regulation and metabolic benefits post-bariatric surgery can be attributed to changes in GLP-1 [185]. Therefore, individuals who achieve an optimal clinical response (total weight loss% > 20%) after gastric bypass exhibit a significant GLP-1 response, while those with poor weight loss outcomes have an attenuated GLP-1 response [186]. Similarly, GLP-1 is an important regulator of glucose homeostasis, as blockage of its receptor hampers its metabolic benefits [187]. It should also be noted that endogenously secreted GLP-1 levels are elevated in both RYGB and SG, with RYGB promoting the higher post-prandial GLP-1 concentrations due to bypassing the proximal small intestine [188].

Distinct gut microbial signatures have been identified in association with GLP-1 and GLP-2 secretion and related control of glucose homeostasis following RYGB, specifically [146]. The Families *Akkermansiacea*, *Rickenellaceae*, *Veillonellaceae*, *Enterobacteriaceae*, and *Rickenellaceae* were positively correlated with hormone secretion, while *Lachnospiraceae* and *Rumminococcacae* showed an opposite effect [146]. In general, microbiota-related processes are shown to be significantly intertwined with the release of GLP-1, as well as GLP-1 receptor (GLP-1R) signaling to influence satiety [182,183]. For example, the depletion of gut microbiota attenuates hypothalamic inflammation induced by a high-fat diet in GLP-1R-deficient murine models, leading to improved leptin sensitivity [183]. Similarly, prebiotic administration attenuated intestinal permeability and inflammation, while promoting the endogenous production of GLP-2 [189].

The mechanisms by which bariatric surgery directly influences increased GLP-1 secretion involve direct and indirect contributions from microbiota metabolites [190]. For example, in rodent models of SG, lithocholic acid has been identified as a microbiota metabolite that remodels the gut–liver axis post-surgically [147]. Specifically, SG-induced shifts in gut microbiota composition upregulate the expression of bile acid transporters, allowing lithocholic acid to be transported across the gut epithelium, as evidenced by increased metabolite concentrations in the portal vein [147]. Therefore, these bile acids may act as a TGR5 agonist, which promotes increased GLP-1 release [109,191]. Transplanting gut microbiota from SG-operated mice to GF mice induced a similar effect, with lithocholic acid serving an important role in GLP-1 secretion [147]. Interestingly, bariatric surgical procedures such as bile diversion to the ileum also promote GLP-1 through the agonism of the FXR receptor, an effect blocked by the GLP-1 receptor antagonist [110,192]. These positive findings were shown to be mediated by *Akkermansia muciniphila*, whose increased abundances were directly correlated with GLP-1 secretion [192]. *Akkermansia* contains an 84kDa protein, termed P9, which interacts with intracellular adhesion molecule-2 to induce thermogenesis in brown adipose tissue and induce systemic GLP-1 release [148]. In addition to bile acid-mediated signaling, gut microbiota influences GLP-1 secretion through the production of SCFA and the resulting activation of GPR43 [193,194]. The infusion of acetate in humans stimulates GLP-1 and PYY release [193], while GPR43 knockout rodent models did not exhibit a similar response to SCFAs [194,195]. PCR quantification demonstrated that SCFA receptor expression is enriched in the intestinal L-cells, with SCFAs raising calcium concentrations and triggering GLP-1 release [194]. Following bariatric surgery in rodent models, it has been shown that increased intestinal SCFA receptor expression, including both GPR41 and GPR43, correlates with increased GLP-1 concentrations observed after intervention [149]. Taken together, there is strong evidence for the impact of bariatric surgery on GLP-1 secretion resulting from changes in gut microbial composition.

### 4.4. Bariatric Surgery, Gut Microbiota, and PYY

PYY, similar to GLP-1 and GLP-2, is secreted from intestinal L-cells post-prandially to induce satiety and reduce food intake by preferentially binding to its receptor, Y2 [196]. Bariatric surgeries, including SG and RYGB, are shown to increase short- as well as long-term PYY concentrations following intervention [144,186,197]. Further, the weight loss following RYGB has been significantly correlated with PYY response following a meal [198]. GLP-1 and PYY often work in tandem to promote these satiation effects, as GLP-1 antagonists caused the compensatory increase in PYY secretion in bariatric surgery patients [199]. The effects of PYY are not limited to the observed weight loss alone, and it is an integral component of the resolution of diabetes and glucose homeostasis post-surgery [200].

The mechanisms of increased PYY secretion following bariatric surgery are multi-fold, including both microbiota-dependent and independent pathways. For example, vagus nerve-dependent communication allows for the sensing of meal consumption prior to the availability of nutrients to intestinal L cells, which stimulates the secretion of PYY [201], likely secondary to anatomical changes, taste receptors, and alterations in related mechanoreceptors. Additionally, these anatomical alterations in RYGB, for example, can cause a concomitant expedited transfer of nutrients to the distal gut, where nutrient-sensing receptors and intestinal L cells reside [202]. At this point, microbiota metabolites are significantly implicated in satiety, as increased SCFA levels, including propionate, butyrate, isobutyrate, and isovalerate, showed a strong correlation with increased PYY levels in rodent models undergoing both SG and RYGB [98]. This phenomenon can be elucidated by conducting additional studies that assess the impact of energy balance on mice lacking GPR41, which notably demonstrate a reduced expression of PYY compared to their wild-type counterparts. As such, the GPR41 nutrient-sensing receptors also detect food composition to stimulate the release of PYY from intestinal L-cells. In addition, restoration of the *Firmicutes*/*Bacteroidetes* ratio has been shown to increase serum PYY levels and serves as another potential mechanism by which bariatric surgery may exert beneficial effects via this satiety hormone. Lastly, recent data have shown that PYY may also exert exocrine anti-microbial effects against pathogenic bacteria [203], which may further contribute to improved gut microbial remodeling post-surgery in a reciprocal fashion.

### 4.5. Bariatric Surgery, Gut Microbiota, and Effects on Orexigenic/Anorexigenic Neuropeptides

**Figure 5:**
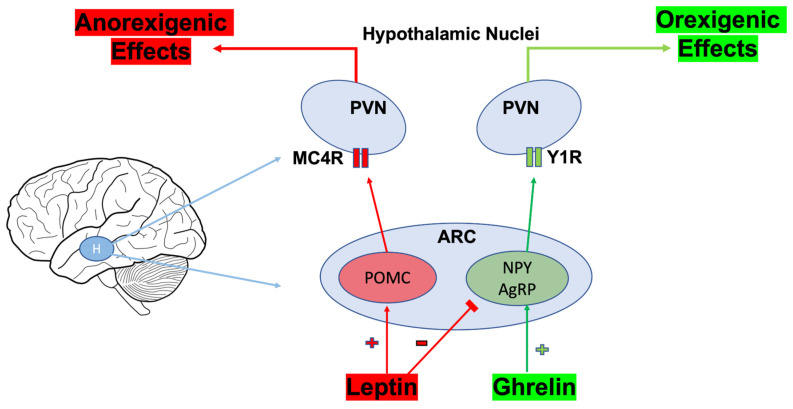
Hypothalamic Network Involving Neuropeptide Y (NPY), Agouti-Related Peptide (AgRP), Proopiomelanocortin (POMC), and Effects on Regulating Appetite. Leptin influences both POMC and NPY/AgRP, with a stimulatory effect on POMC and inhibitory effect on NPY/AgRP neurons within the arcuate nucleus of the hypothalamus. Ghrelin, on the other hand, stimulates NPY/AgRP neurons. POMC neurons in the arcuate nucleus project to the paraventricular nucleus where its receptor, melanocortin receptor 4 (MC4R), resides. Upon activation of MC4R, anorexigenic signals are sent to the periphery. NPY/AgRP neurons, once stimulated, also can project to the paraventricular nucleus and activated NPY receptor, Y1, sending orexigenic signals to the periphery. Abbreviations: H, hypothalamus; MC4R, melanocortin receptor 4, PVN; paraventricular nucleus; POMC, proopiomelanocortin; ARC, arcuate nucleus; NPY, neuropeptide Y; AgRP; agouti-related peptide; Y1R, Y1 receptor.

The hypothalamic arcuate nucleus (ARC) contains two subgroups of neurons that work in an antagonistic manner and are activated by nutrient-sensing and gut hormones, among other factors [204]. One group of neurons, AgRP and NPY, are orexigenic and can be co-expressed, while the other group involves the anorexigenic neuropeptides, POMC and CART [205,206]. Bariatric surgery alters the expression of these neurons, possibly by preventing biologically adaptive behavior to release orexigenic neuropeptides stimulated by undernutrition [207]. Unlike calorie restriction, AgRP and NPY expression is not increased following surgical intervention, while markers of neuroinflammation and oxidative stress were concurrently reduced, including the ionized calcium-binding adaptor molecule (Iba1), which is a marker of microglial activity [207,208]. Although the exact mechanisms are unclear, leptin, ghrelin, PYY, and other gut peptides directly act within the ARC to stimulate the expression of these neuropeptides (i.e., NPY and AgRP); therefore, the altered circulating hormone levels may contribute to these observed changes to affect energy balance following surgery [209]. By contrast, obesity inhibits the ARC-mediated modulation of energy balance due to related and sustained neuropeptide release in response to hunger [209] (Figure 5). This is evidenced by studies using diet-induced obesity mice undergoing RYGB, with improvements in vagal neuronal responses in the DMV and restored responses to gut peptides following the surgery [150]. Given that bariatric surgery confers changes in neuronal plasticity, there seems to be a surgical influence on these processes, including the microbial contribution via the microbiota–gut–brain axis.

Our recent data has shown that GF mice had an increased NPY and AgRP mRNA in the hypothalamus and a decrease in the hindbrain [123]. Both the administration of leptin and re-introduction of microbiota normalized AgRP and NPY mRNA, indicating a significant role for microbiota in these processes. Of note, the mRNA expression of anorexigenic neuropeptides, POMC and CART, was not significantly different between the GF and conventionally raised mice [123], though similar studies have shown reductions in hypothalamic POMC and CART mRNA levels. Increases in microbiota metabolites, notably acetate, suppress NPY and AgRP expression by attenuating gamma-aminobutyric acid (GABA) neuroglial activity [151]. Notably, our recent study suggests that altered GABA signaling may ensue from RYGB affecting extrahypothalamic areas playing a role in memory and perception, all likely to influence eating behaviors [207]. GABA is shown to be either co-expressed with NPY/AgRP neurons or act on other GABA-ergic neurons to promote feeding behaviors [210]. Reductions in GABA or administration of GABA antagonists promote an opposite effect, lessening the orexigenic neuropeptide activity [210]. Further, hypothalamic inflammation observed in states of obesity may significantly influence the expression of these neuropeptides, which is reversed following bariatric surgery with the associated normalization of NPY and AgRP when compared to lean counterparts [85]. NPY has also been shown to exert a reciprocal role on microbiota composition by demonstrating antimicrobial effects against *Escherichia coli*, *Enterococcus*, and *Lactobacillus* [211] through the regulation of innate and adaptive immune processes [212]. It is also important to note that NPY and PYY are part of the same biologically active family that acts on similar receptors, Y1–Y5, with PYY exerting inhibitory effects on NPY and the subsequent activation of POMC neurons [213]. The nodose ganglion and vagal afferents are essential in conveying information via gut-specific PYY projections to ARC neurons [214]. Therefore, the microbiota’s effects on PYY expression following bariatric surgery are an important factor contributing to altered hunger and satiety.

## 5. Bariatric Surgery, Gut Microbiota, Dopamine, and Reward Pathways

In addition to altering levels of gut hormones and neuropeptides, bariatric surgery mediates weight loss through the modulation of reward pathways and, in turn, behaviors associated with the hedonic control of eating [215,216]. In a recent meta-analysis, it was shown that the prevalence of food addiction of individuals pre-bariatric surgery was 32%, compared to 15% after surgery [153] (Table 1). Further, neural predictors of estimated total weight loss following bariatric surgery are shown to be dependent on the baseline activity in the nucleus accumbens and hippocampus during the desire for palatable food [217]. One study has shown that SG and RYGB induce similar reductions in hedonic hunger when compared to controls fed a low-energy diet [215]; however, RYGB has been demonstrated to improve the neural processing of reward stimuli in response to sweet foods to a greater extent than SG [218]. Dopamine mediates these behavioral changes with obese individuals exhibiting brain reward deficits in response to palatable foods, leading to increased consumption to achieve the same level of activation in involved regions of the brain as lean counterparts [219]. More specifically, dopaminergic signaling pathways, such as mesolimbic, mesocortical, and nigrostriatal pathways, are affected in obesity with decreased excitability and down-regulated dopamine receptor and dopamine transporter (DAT) expression within the nucleus accumbens, ventral tegmental area (VTA), and the striatum as obesity progresses [219,220].

Gut microbiota are intertwined with these neurophysiological processes, as recently reviewed [22]. For example, in a study assessing the gut microbial profiles of obese women with food addiction, altered levels of bacterial genera *Bacteroides*, *Eubacterium*, and *Akkermansia* were associated with reward behaviors linked to feeding [221]. Indoleproprionic acid, a microbiota metabolite with neuroprotective antioxidant effects, was identified to be inversely correlated with food addiction. Increased diversity and richness of gut microbial species, including butyrate-producing species such as *Eubacterium* are shown to be associated with indoleproprionic acid concentrations [222]. Therefore, with states of dysbiosis, microbial diversity is reduced along with important metabolites including indoleproprionic acid that may contribute to food addiction. Further, *Parabacteroides*, a genus associated with aggravating enteroendocrine signaling, is positively correlated with a high-fat diet intake and hedonic food behaviors [223]. Previous studies also underscored the significance of gut microbiota in these mechanisms, as evidenced by an increased preference for palatable foods after an antibiotic-induced depletion of gut microbiota. Likewise, the transplantation of microbiota from obese mice into lean recipients replicated the dysregulation of hedonic food intake behavior [223]. However, it should be noted that even with direct inoculation of specific bacteria after antibiotic depletion in recipient mice, dietary habits such as the consumption of a high-fat or a Western diet confer more sustained alterations in gut microbial composition [224]. Additionally, optimizing gut microbiota beyond just surgically induced changes may protect against food addiction following bariatric surgery [225]. For example, a recent study has shown that probiotic supplementation containing *Lactobacillus* and *Bifidobacterium* spp. for 90 days post-operation decreased binge eating behaviors for up to one year when compared to controls undergoing RYGB [225]. It should be noted that the data on preventing recurrent weight gain or promoting further weight loss with probiotic supplementation after bariatric surgery do not show superiority when compared to control groups [226,227,228]. There are ongoing clinical studies to further characterize the effects of probiotic supplementation on food addiction as well as satiety peptides, which will provide further insight into this topic [229].

Considering the substantial impact of gut microbiota on dopaminergic circuits associated with food addiction, it is conceivable that alterations in microbial composition induced by bariatric surgery could significantly contribute to the improvements observed in reward-related behaviors following the intervention. For example, SG promoted improvements in maladaptive eating behaviors, with alterations in *Bacteroides*, *Ruminococcus*, and *Holdemanella* being associated with reduced resting-state connectivity in the amygdala and putamen [154]. Of interest, a microbiota metabolite derived from phosphatidylcholine, 1-palmitoyl-2-palmitoleoyl, was found to be implicated in these processes, as positive associations were observed with its concentrations and the resting state connectivity between the putamen and precuneus [154]. Further, in obese individuals, the *Prevotella*/*Bacteroides* ratio was elevated, an effect that was positively associated with nucleus accumbens centrality, which is the brain reward center [124]. As previously mentioned, individuals with a high *Prevotella*/*Bacteroides* ratio lost significantly more weight after bariatric surgical interventions, indicating a role in the brain reward systems and dopamine processing areas [19]. These specific pathways and respective brain regions associated with food addiction, gut microbiota, and bariatric surgery will be discussed in further detail in the following subsections.

### 5.1. Bariatric Surgery, Gut Microbiota, Mesolimbic Pathway, and Striatal Dopamine

The mesolimbic dopaminergic system is known to mediate reward, therefore playing a significant role in food-seeking behaviors [230]. In this pathway, dopamine is transported from the ventral tegmental area primarily to the nucleus accumbens within the ventral striatum, but also to the amygdala and hippocampus [230]. Gut microbiota and their metabolites are one of the factors that can influence mesolimbic dopamine, its receptors, and transporters to promote weight gain. Primarily, this occurs through microbiota–gut–brain signaling via vagal afferents. When the microbiota of high-fat-diet-induced mice were colonized into germ-free mice, lower dopamine metabolite levels and D2 receptor expression in the nucleus accumbens compared to low-fat-fed counterparts were observed [231]. In addition, dopamine metabolite levels and dopamine receptor expression were restored following the selective vagotomy of gut–brain afferents in these same mice [231]. Importantly, we have shown that RYGB restored high-fat diet effects on the dopamine system such as the availability of dopamine 2 receptor (D2R), dopamine 1 receptor (D1R), and dopamine transporter (DAT) binding in the striatum [232]. Neuroactive SCFAs may act on vagal afferents to directly, or indirectly, influence mesolimbic dopamine [233]. For example, increased propionate production attenuates reward-based eating behaviors in the caudate and nucleus accumbens [233].

Obese patients undergoing SG had an increase in *Akkermansia* that was correlated with reduced hedonic eating behaviors [155]. Although this study did not include RYGB patients, this surgery also promotes increased *Akkermansia*, which may confer similar benefits. The influence of *Akkermansia* on food reward processes has been described in another study, where *Akkermansia* was administered to diet-induced obese mice [156]. After supplementation, dysregulated reward behaviors were improved through reductions in striatal inflammation and blood–brain barrier permeability [156]. As previously discussed, metabolic endotoxemia secondary to perturbations in microbiota leads to the low-grade systemic inflammation seen in obesity, including neuroinflammation, often through excessive activation and maturation of microglia [82,234]. Systemic inflammation not only alters the expression of gut hormones and related neuropeptides but is also implicated in dopamine bioavailability and eating behaviors. For example, the LPS-induced activation of TLR-4 contributes to microglial activity within the VTA, thereby increasing extracellular dopamine in the nucleus accumbens shell to influence reward pathways [157] (Table 1). Further, murine models of high-fat-induced obesity exhibit both hyperleptinemia as well as nucleus accumbens-specific inflammation, provoking compulsive sucrose-seeking feeding behaviors [235]. Such a behavioral response was promoted through an increased nuclear factor of kappa beta (NF-κB) transcriptional activity. NF-κB activation initiates a systemic inflammatory response through the expression of associated pro-inflammatory cytokines, chemokines, and inflammatory T-cells in response to diverse stimuli including certain strains of microbiota and their metabolites [236,237]. *Akkermansia* has been shown to suppress NF-κB activation through the stimulation of TLR2, thereby mitigating increased inflammation and improving reward-related behaviors. Additionally, *Akkermansia* is decreased in individuals with binge eating behaviors, thereby further supporting its importance in eating behaviors.

*Bacteroides* is also increased in RYGB patients and has been associated with both food addiction and striatal dopamine [238,239]. Hartstra et al. have shown that *Bacteriodes uniformis* was significantly associated with increased DAT binding, while *Prevetolla* spp. was inversely correlated with the same parameter. DAT packages extracellular dopamine into synaptic vesicles so that dopamine may be re-released into the synaptic cleft [158]. In obesity, DAT binding is reduced in the striatum [232]; therefore, less dopamine is available within synaptic clefts during palatable food consumption [240]. As discussed above, our previous work showed that RYGB in the fat-diet-induced obese rat model restored blunted DAT availability [232]. *Bacteroides uniformis* modulates hedonic eating behaviors by improving dopaminergic expression in the nucleus accumbens as well as receptor expression in the prefrontal cortex in a rat model of food addiction. Taken together, these studies provide strong evidence for the role of *Bacteroides*, an important genus that is elevated post-RYGB, in improving parameters of dopaminergic signaling as it relates to reward processing. The differential findings between *Prevotella* and *Bacteroides* on DAT binding provide further insight into the benefits of post-bariatric surgery-induced reductions in the *Prevotella*/*Bacteroides* ratio discussed throughout this review.

### 5.2. Bariatric Surgery, Gut Microbiota, Dopamine, and Gut Hormones

Numerous mechanisms involved in hedonic responses to food consumption are directly associated with changes in gut hormones and the corresponding effects on microbiota and their metabolites [241]. For example, ghrelin alters dopaminergic receptor expression by acting on its receptor, GHSR1a, in the VTA and nucleus accumbens [242]. GHSR1a activation leads to dimerization with various receptors including D1R and D2R leading to down-stream signaling. The VTA acts as a convergence point between the ghrelinergic and dopaminergic systems, mediating reward processing in response to food intake [242,243]. Specifically, an increase in ghrelin concentrations induces dopamine-related gene expression, stimulates dopamine release, and increases food intake-related behaviors and impulsivity [244]. Similarly, central ghrelin receptor antagonist administration reduces these behavioral effects and dopamine release within the VTA and nucleus accumbens [245,246]. As such, the microbiota-related changes that increase ghrelinergic signaling discussed in a previous subsection may also indirectly promote impulsive food-seeking behaviors, dopamine release, and recruit dopaminergic receptors. Further, the presence of microbiota metabolites, particularly SCFA, attenuates ghrelin receptor signaling, thereby supporting the role of SCFA-producing genera in improving these processes [168]. At the same time, bariatric surgery mitigates these effects, with post-RYGB murine models exhibiting reduced tonic dopamine firing secondary to diminished GHSR1a activity [159]. These findings are correlated with decreased ghrelinergic signaling through improvements in the *Firmicutes*/*Bacteroidetes* ratio [160].

Leptin receptors are also expressed in the VTA and substantia nigra [247]. In the midbrain, leptin activates these receptors to promote JAK-STAT3-induced downstream signaling, reducing VTA and mesolimbic dopamine neuronal firing rates [248]. This concurrently lessened food-motivated and reward behaviors [248,249]. By contrast, STAT3-deficient mice have reduced mesolimbic dopamine function, requiring more stimulation to achieve a similar food-related reward [250]. Administration of prebiotics promotes an increased leptin expression in the nucleus accumbens with reduced DAT expression in the VTA [251]. Bariatric surgery decreases leptin, an effect associated with microbiota metabolites and improvements in microbial composition [252]. Leptin acts synergistically to improve *Bacteroides* concentrations and overall bacterial diversity [253], although supplementation of *Bacteroides* has also been shown to have a beneficial effect on leptin [254], both of which are improved following bariatric surgery. Although the direct investigation of how gut microbiota, particularly post-bariatric surgery, influences leptin to enhance reward behaviors has not been directly studied, evidence exists supporting the influence of microbiota on striatal dopamine receptors, which can be influenced by surgical interventions [255].

GLP-1, PYY, and CCK are also highly involved in reward-mediated effects of bariatric surgery and associated weight loss. It has been well-described that the activation of GLP-1 receptors in the NTS contributes to food reward-suppressing behaviors through targeting mesolimbic dopamine [161,256]. Similar findings have been observed in the striatum following the activation of the Y2 receptor by PYY [257], as well as CCK receptor activation, with CCK-1R-deficient obese rats displaying reduced basal dopamine transmission [240]. Interestingly, several studies have shown that SG-induced increases in *Clostridia* may initiate changes in the striatum to improve GLP-1 release [258]. *Clostridium* spp., particularly *Clostridium butyricum* and *symbiosum*, have been shown to promote GLP-1 secretion irrespective of surgical intervention type [259,260], therefore potentially contributing to changes in mesolimbic dopamine activity as well. It is also shown that different surgical methods alter these food-seeking behaviors to various degrees, with RYGB having a more significant effect than gastric banding [241]. For example, gastric bypass patients exhibited a lower activation of reward systems in response to high-calorie foods within the mesolimbic system including amygdala, nucleus accumbens, and hippocampus. In conclusion, the intricate interplay between bariatric surgery, gut microbiota, dopamine, and various gut hormones highlights a complex mechanism through which these factors collectively influence hedonic responses to food, impulsive food-seeking behaviors, and ultimately, weight loss.

## 6. Conclusions and Perspectives

There is significant evidence supporting the central role of gut microbiota in processes that contribute to weight loss following bariatric surgery. The intricate interplay between bariatric surgery, gut microbiota, dopamine signaling, and gut hormones encompasses several key mechanisms that contribute to the procedure’s effectiveness in promoting weight loss and improving eating behaviors. Overall, bariatric surgery leads to significant changes in the composition of the gut microbiota, including shifts in the *Firmicutes*/*Bacteroidetes* ratio and increases in beneficial genera such as *Akkermansia*. These changes are associated with improved metabolic outcomes and may influence eating behaviors by modulating the gut–brain axis. In addition, bariatric surgery affects the levels of various gut hormones that play critical roles in hunger and satiety. Ghrelin, leptin, GLP-1, PYY, and CCK are modulated post-surgery, contributing to reduced appetite, increased feelings of fullness, and altered food preferences. Furthermore, surgery-induced alterations in gut microbiota can affect dopamine signaling pathways, which are crucial for regulating reward processing and hedonic eating. By modifying dopamine receptor expression and dopamine release within key brain regions like the VTA and nucleus accumbens, bariatric surgery can reduce compulsive food-seeking behaviors and impulsivity. Further investigations are warranted into the underlying mechanisms by which bariatric surgery alters the gut microbiota and its impact on neural and hormonal pathways related to food intake and reward, as they are expected to offer deeper insights into the interconnections between metabolism, gut health, systemic inflammation, and brain function. The development of non-surgical interventions that mimic the beneficial effects of bariatric surgery on the gut microbiome and hormonal regulation offers a promising avenue for treating obesity without the need for invasive procedures. For example, exploring the potential of microbiota-targeted interventions, such as prebiotics, probiotics, and fecal microbiota transplantation, as adjunct therapies to bariatric surgery could pave the way for personalized treatment strategies.

## Figures and Tables

**Table 1:** Gut Microbiota-Mediated Effects on Satiety Peptides, Neuropeptides, and Dopamine Following Bariatric Surgery.

Satiety Hormone, Neuropeptide or Neurotransmitter	Species Involved/Outcome Measured	Results/Implications	Humans vs. Murine	References
Leptin	Serum SCFA	In SG, leptin levels are reduced in correlation with upregulation of SCFA and increased expression of colonic GPR43.	Mice	[52]
	Inflammatory Markers	In obesity, LPS- or LTA-mediated low-grade inflammation contributes to diminished sensitivity of the NG to leptin activity. RYGB mitigates these effects.	Rats	[78]
	Serum Leptin	Bariatric surgery lowers serum leptin levels to alleviate leptin resistance.	Humans	[133]
	*Escherichia* *Bacteroides*	RYGB-mediated alterations correlate with serum leptin and fat mass. Increased *Bacteroides* is associated with lower serum leptin.	Humans	[137]
	Hypothalamic Inflammation	Reduction in leptin inhibitor, SOCS3, following bariatric surgery mitigates leptin resistance. Microbiota-related changes improve hypothalamic inflammation.	Humans	[138]
Ghrelin	Serum Ghrelin	Immediately after surgery, ghrelin levels are decreased in SG and increased in RYGB. At one year, both surgeries decrease ghrelin concentrations.	Humans	[135]
	*Faecalibacterium*	Elevated following RYGB and negatively correlated with ghrelin concentrations. SCFA-producing bacterium promotes downstream inhibitory effects on ghrelin secretion following GPR43 binding.	Humans	[137]
	Inflammatory Markers	LPS activation of TLR-4 converges with activity of the GHR1a receptor. States of metabolic endotoxemia may modulate ghrelinergic activity. Bariatric surgery mitigates metabolic endotoxemia.	Mice	[139]
	Hydrogen Sulfide Concentrations	H2S suppresses ghrelinergic signaling via GHR1a receptor. H2S prolongs post-prandial drop of serum ghrelin and reduced food intake. RYGB promotes increased abundance of *Escherichia*, *Veillonella*, *Clostridium*, *Bacteroides*, and *Streptococcus*, all of which have H2S-producing capabilities.	Mice	[140]
Cholecystokinin	Serum CCK	Following bariatric surgery, CCK receptor expression is increased post-prandially.	Humans	[141]
	Endocannabinoids	Endocannabinoid concentrations are characteristically decreased after bariatric surgery. Sustained weight loss in RYGB correlates with reduced endocannabinoids and related increases in serum CCK.	Rats	[142]
	SCFA	GPR41 expression in the NG is essential for CCK secretion. Improved SCFA profile following bariatric surgery enhances its secretion.	Mice	[143]
Glucagon-Like Peptide 1	GLP-1 Concentrations	GLP-1 concentrations are increased as early as 1-week post-surgical intervention; GLP-1 remains elevated 10 years post-surgery.	Humans	[144,145]
	Gut Microbiota	*Akkermansiacea*, *Rickenellaceae*, *Veillonellaceae*, *Enterobacteriaceae*, and *Rickenellaceae* were positively correlated with GLP-1, while *Lachnospiraceae* and *Rumminococcacae* had an opposite effect. These changes are evident in RYGB.	Humans	[146]
	Bile Acids	Lithocholic acid is elevated following SG; acts as agonist for TGR5 to influence GLP-1 secretion.	Rodent	[147]
	*Akkermansia*	*Akkermansia* is elevated after SG and RYGB; the protein component, P9, interacts with ICAM-2 to induce thermogenesis and systemic GLP-1 release.	PCR	[148]
	SCFA	Acetate infusions trigger GLP-1 release through raising calcium concentrations. In bariatric surgery, GPR41 and GPR43 expression is increased, correlating with enhanced GLP-1 secretion.	Rodent	[149]
Peptide YY	SCFA	SCFA profile changes in bariatric surgery; propionate, butyrate, isobutyrate and isovalerate correlate with increased PYY in both SG and RYGB.	Rodent	[98]
	PYY Concentrations	SG and RYGB increase PYY concentrations.	Humans	[144]
Orexigenic and Anorexigenic Neuropeptides	Hypothalamic Inflammation	Bariatric surgery reverses hypothalamic inflammation and normalizes NPY and AgRP.	Mice	[85]
	Neuropeptides	RYGB improves vagal neuronal health in the DMV to restore neuropeptide response to gut hormones.	Rats	[150]
	NPY and AgRP	Acetate suppresses NPY and AgRP expression by attenuating GABA activity. GABA signaling ensues following RYGB-induced alterations in extrahypothalamic areas that integrate memory and perception, all influencing eating behaviors.	Mice	[151,152]
Eating Behaviors	Food Addiction	Bariatric surgery reduces prevalence of food addiction by 17%.	Humans	[153]
	Eating Behaviors	SG improved maladaptive eating behaviors, which are associated with altered concentrations of *Bacteroides*, *Ruminococcus*, and *Holdemanella*. Microbiota metabolite 1-palmitoyl-2-palmitoleoyl is associated with reduced resting-state connectivity in amygdala and putamen.	Humans	[154]
	Hedonic Eating	SG elevated *Akkermansia* in obesity; was correlated with reduced hedonic eating. *Akkermansia* improves dysregulated reward behaviors through reductions in striatal inflammation and blood–brain barrier permeability.	Humans	[155,156]
Dopamine	Extracellular Dopamine	LPS-induced activation of TLR-4 contributes to microglial activity within the VTA. Extracellular DA increases in NAcc shell. Bariatric surgery attenuates LPS-mediated inflammation.	Rodent	[157]
	DAT	*Bacteroides uniformis* is associated with increased DAT binding, while *Prevotella* spp. has an opposite effect.	Rodent	[158]
	Ghrelin/Dopamine	Post-RYGB murine models exhibit reduced tonic dopamine firing secondary to diminished GHSR1a activity. Decreased ghrelinergic signaling with concomitant improvements in the *Firmicutes*/*Bacteroidetes* ratio.	Rodent	[159,160]
	GLP-1/Dopamine	Activation of GLP-1 receptors in the NTS contributes to food reward suppressing behaviors through targeting mesolimbic DA.	Rats	[161]

Abbreviations: RYGB, Roux-en-Y gastric bypass; LPS, lipopolysaccharides; LTA, lipoteichoic acid; NG, nodose ganglion; SG, sleeve gastrectomy; SCFA, short-chain fatty acids; GPR43, G-coupled protein receptor 43; SOCS3, suppressor of cytokine signaling 3; TLR-4; toll-like receptor 4; H2S, hydrogen sulfide; GHSR1a, growth hormone secretagogue receptor; GPR41, G-coupled protein receptor 41; CCK, cholecystokinin; GLP-1, glucagon-like peptide 1; TGR5, Takeda G protein-coupled receptor 5; ICAM-2, intracellular adhesion molecule 2; PCR, polymerase chain reaction; PYY, peptide YY; NPY, neuropeptide Y; AgRP, agouti-related peptide; DMV, dorsal motor nucleus of vagus; GABA, gamma-aminobutyric acid; DAT, dopamine transporter; VTA, ventral tegmental area; DA, dopamine; NTS, nucleus of tractus solitarius.
